# Supplementary material for: Long-Term Medical Resource Consumption between Surgical Clipping and Endovascular Coiling for Aneurysmal Subarachnoid Hemorrhage: A Propensity Score–Matched, Nationwide, Population-Based Cohort Study
Source: Int J Environ Res Public Health. 2021 Jun 2;18(11):5989. doi: 10.3390/ijerph18115989 (PMC8199713; doi:10.3390/ijerph18115989)
Supplement: Supplementary file 1 [file ijerph-18-05989-s001.zip › ijerph-1213219-supplementary.pdf]

Table S1. Demographic and clinical parameters of patients with ruptured intracranial aneurysms before propensity score–matched.

		**Endovascular coil embolization N=4,700**		Surgical clipping N=6,109		*P* Value
		**n**	(%)	n	(%)	
Age	Mean (SD)	57.9	(13.9)	56.9	(13.0)	<0.0001
	Median (Q1-Q3)	58	(49 - 68)	57	(48 - 66)	
	20-64	3163	(67.3)	4399	(72.0)	<0.0001
	65-74	894	(19.0)	1092	(17.9)	
	75-84	544	(11.6)	556	(9.1)	
	85+	99	(2.1)	62	(1.0)	
Gender	Male	1741	(37.0)	2238	(36.6)	0.6628
	Female	2959	(63.0)	3871	(63.4)	
Treatment year	2011	422	(9.0)	922	(15.1)	<0.0001
	Male	155	(3.3)	338	(5.5)	
	Female	267	(5.7)	584	(9.6)	
	2012	471	(10.0)	879	(14.4)	<0.0001
	Male	175	(3.7)	321	(5.3)	
	Female	296	(6.4)	558	(9.1)	
	2013	566	(12.0)	852	(13.9)	0.2031
	Male	209	(4.4)	312	(5.1)	
	Female	357	(7.6)	540	(8.8)	
	2014	668	(14.2)	899	(14.7)	0.5685
	Male	247	(5.2)	321	(5.2)	
	Female	421	(9.0)	578	(9.5)	
	2015	699	(14.9)	848	(13.9)	0.3684
	Male	259	(5.5)	303	(5.0)	
	Female	440	(9.4)	545	(8.8)	
	2016	904	(19.2)	878	(14.4)	<0.0001
	Male	335	(7.1)	313	(5.2)	
	Female	569	(12.1)	565	(9.2)	
	2017	970	(20.6)	831	(13.6)	<0.0001
	Male	361	(7.7)	330	(5.4)	
	Female	609	(12.9)	501	(8.2)	
Location of aneurysm	ACA	2309	(49·1)	2632	(43·1)	<0.0001
	ICA	576	(12·3)	635	(10·4)	
	MCA	1372	(29·2)	2058	(33·7)	
	VBA	302	(6·4)	484	(7·9)	
	PCA	141	(3·0)	300	(4·9)	
Diabetes	No	3992	(84.9)	5338	(87.4)	0.0002
	Yes	708	(15.1)	771	(12.6)	
Congestive heart failure	No	4576	(97.4)	5994	(98.1)	0.0081
	Yes	124	(2.6)	115	(1.9)	
Hypertension	No	2268	(48.3)	3119	(51.1)	0.0039
	Yes	2432	(51.7)	2990	(48.9)	
Renal diseases	End-stage renal disease	47	(1.0)	52	(0.9)	0.0549
	Chronic kidney disease	157	(3.3)	159	(2.6)	
	No renal diseases	4496	(95.7)	5898	(96.5)	
Stroke or TIA	No	2678	(57.0)	4445	(72.8)	<0.0001
	Yes	2022	(43.0)	1664	(27.2)	
CCI Scores	0	631	(13.4)	1970	(32.2)	<0.0001
	1	2771	(59.0)	2931	(48.0)	
	2+	1298	(27.6)	1208	(19.8)	
Hospital level	Academic centers	3809	(81.0)	4255	(69.7)	<0.0001
	Nonacademic centers	891	(19.0)	1854	(30.3)	
Hospital area	North	2929	(62.3)	2625	(43.0)	<0.0001
	Center	738	(15.7)	1779	(29.1)	
	South	915	(19.5)	1510	(24.7)	
	East	118	(2.5)	195	(3.2)	
Income	<NTD 18,000	1062	(22.6)	1344	(22.0)	<0.0001
	NTD 18,000-22,500	929	(19.8)	1554	(25.4)	
	NTD 22,500-30,000	1068	(22.7)	1257	(20.6)	
	NTD 30,000+	1641	(34.9)	1954	(32.0)	
Follow-up time, months	Mean (SD)	54.2	(28.1)	50.1	(25.6)	<0.0001
	Median (Q1-Q3)	53.2	(31.2 - 78.1)	49.0	(30.2 - 69.1)	
All-Cause Death		811	(17.3)	1366	(22.4)	<0.0001
